# Body Dissatisfaction, Distorted Body Image and Disordered Eating Behaviors in University Students: An Analysis from 2017–2022

**DOI:** 10.3390/ijerph191811482

**Published:** 2022-09-13

**Authors:** Karime Jiménez-Limas, Verónica Anayansi Miranda-Barrera, Karla Fernanda Muñoz-Díaz, Samantha Ruth Novales-Huidobro, Gabriela Chico-Barba

**Affiliations:** 1 School of Nursing, Faculty of Health Sciences, Universidad Panamericana, Mexico City 03920, Mexico; 2 Nutrition and Bioprogramming Coordination, Instituto Nacional de Perinatología, Mexico City 11000, Mexico

**Keywords:** Body Dissatisfaction, Distorted Body Image, Disordered Eating Behaviors, Risky Eating Behaviors, Body Image, Desired Body Image, Body Percept, Body Perception, Mexico, Young Adulthood

## Abstract

University students, as young adults, are at risk for Body Dissatisfaction (BD) and Distorted Body Image (DBI), which are related to Disordered Eating Behaviors (DEBs). This study aimed to assess changes in the prevalence of these three conditions over six years; and the associations between them. Data was collected through an annual online survey from 2017 to 2022 at a private university in Mexico City. Students between 18–30 years old were invited to participate. Body image-related variables were assessed by the Stunkard’s Silhouettes and Body Mass Index, by self-reported height and weight. Disordered Eating Behaviors were measured by the Brief Disordered Eating Behaviors Questionnaire. A median of 250 students participated per year, with a median age of 21 years old. The prevalence was 63.5–71.7% for BD, 40.4–49.1% for DBI, and 25–38.3% for DEBs. DEBs and BD showed associations during the whole period (OR from 3.6 to 15.9, *p* ≤ 0.001); as well as DBI with DEBs (OR from 1.9 to 3.3, *p* < 0.05). Alterations in Body Image and eating behaviors are common conditions, mainly in women and in the young population. Therefore, it is important to promote screening for these conditions, as they usually remain undiagnosed, their prevalence is increasing worldwide, and their impact on physical and mental health has already been acknowledged.

## 1. Introduction

### 1.1. Body Image-Related Variables

Body Image consists of a set of three complex intentional dimensions, in which the object of such dimensions is one’s own body: Body Percept, which refers to the subject’s total or partial perception of her/his own body; Body Concept, which refers to the conceptual understanding about one´s body (including folk, mythical and scientific knowledge); and Body Affect, which refers to the subject´s attitude towards her/his own body [1]. The main dimension referred to when assessing Body Image is Body Percept (BP). A person´s BP may differ from the Body Image she or he wishes they had; this is known as Body Dissatisfaction (BD) [2]. Meanwhile, as BP is not necessarily linked to the actual physical composition, there can also be a discrepancy between these two, known as Distorted Body Image (DBI) [3,4,5].

In a study carried out in the United States, more than a thousand participants were followed over 15 years, and it was found that Body Dissatisfaction increased over time in both sexes. By the time they entered Young Adulthood, half of the females (50.0%) and more than a quarter of the males (25.9%) were dissatisfied with their bodies. The prevalence of having at least one eating, activity, or weight-related problem increased from the first measurement (in adolescence) to the last one (in adulthood); in females, it escalated from 78.1% to 82.3% and in males, from 60.1% to 69.2% [6]. Other studies have shown a general prevalence of almost 50% to 68.3% for BD [7,8]; from 67.6% to 86.1% in women and 48.5% to 67% in men [8,9]. As for DBI, the prevalence ranges from 50.4% to 59% (for women 48.3–57.5% and for men 51.7–62.1%) [8,10]. This demonstrates that alterations in Body Image represent a public health concern among this population, especially for women. Body Dissatisfaction and Distorted Body Image are considered risk factors for developing Disordered Eating Behaviors (DEBs), especially BD [11]. These three conditions occur particularly in Young Adulthood [12] and university students are at high risk of developing them [13].

### 1.2. Disordered Eating Behaviors and Eating Disorders

DEBs encompass a set of conducts and practices in which food intake is altered, such as binge eating, restrictive diets, the use of laxatives, diuretics, anorexigenics, and/or enemas, the practice of excessive exercise, and self-induced vomiting; all of which have the intention of weight loss, but do not meet the diagnostic criteria for Eating Disorders (EDs) [14]. This is why DEBs are considered to be a condition between two ends, where “normal” eating behavior is on one side and EDs on the other [15]. The onset of DEBs manifests as small-scale voluntary risky conduct, which increases over time, leading to a loss of control over the conduct.

General reported prevalence for DEBs in the United States is 13% in young women and 7% in young men [16] but it has been seen to vary between those with low or standard weight (15.8% for women and 7.5% for men) and those who are overweight or obese (29.3% for women and 15.4% for men) [12].

It is not clear yet if Disordered Eating Behaviors should be considered a preliminary stage or a subclinical condition for Eating Disorders; either way, it is well known that DEBs are considered an important risk factor for EDs [14].

EDs are defined as a group of disorders characterized by physiological and psychological disturbances in appetite or food intake [17]. There are eight of them: Pica, Rumination Disorder, Avoidant/Restrictive Food Intake Disorder, Anorexia Nervosa, Bulimia Nervosa, Binge Eating Disorder, Other Specified Feeding or Eating Disorder and Unspecified Feeding or Eating Disorder. Each one of these is well explained and has specific criteria for its diagnosis, described in the DSM-5 [18,19]. The three most common ones in Young Adulthood are Anorexia Nervosa, Bulimia Nervosa and Binge Eating Disorder [18,19].

A person with Body Dissatisfaction, Distorted Body Image and/or Disordered Eating Behaviors is at high risk for Eating Disorders [11]; if any of these are not detected and treated, they continue progressing over the years [6]. Furthermore, these conditions have been associated with depression, substance abuse (alcohol and tobacco use), malnutrition and significant weight gain over time [18]. It is key to detect BD, DBI and DEBs in order to stop their evolution.

For the reasons mentioned above, the aim of this study was to determine the prevalence (and its changes over a six-year period time) of Body Dissatisfaction, Distorted Body Image and Disordered Eating Behaviors in a sample of Mexican university students. In addition, the associations between them were studied.

## 2. Materials and Methods

### 2.1. Study Design and Sample Collection

Observational, longitudinal research. Data was collected from 2017 to 2022 by an annual online survey using SurveyMonkey^®^. Sampling was convenient, non-probabilistic, and based on consecutive cases that met the following inclusion criteria: aged between 1830 years of age and official enrolment at the undergraduate level in a private university in the south of Mexico City. Students who had incomplete questionnaires were eliminated from the study. This project was approved by the Institutional Review Board and Ethics Committee. All participants gave voluntary consent to be part of the study and their information remained anonymous, as their names were replaced by IDs. The final sample varied according to each year. The 2020 data collection occurred during January and February, before the COVID-19 pandemic had its onset in Mexico.

### 2.2. Sociodemographic and Academic Information

Information regarding age, sex and scholarly year was obtained through a questionnaire.

### 2.3. Body Image-Related Variables

The Stunkard’s Silhouettes were used to evaluate Body Image-related variables. This instrument consists of nine body figures or silhouettes, of both women and men, that go progressively from the thinnest to the thickest body complexion; a number from 1–9 is assigned to each silhouette respectively [20]. Participants had to choose the figure that they believed best represented their actual Body Image and the figure that best represented the Body Image they would like to have; they were allowed to select the same silhouette in both questions. From this instrument we assessed the next four variables.

#### 2.3.1. Body Percept

Body Percept corresponded to the first selected silhouette, the one the participants considered to best represent their actual Body image. As the Stunkard´s silhouettes have proven to be valid representations of the Body Mass Index (BMI) categories [21], a number from 1–9, assigned to each silhouette, was used to link this variable to the BMI categories. The cut points were: 1 as low weight (BMI < 18.5), 2–4 as standard weight (BMI 18.5–24.9), 5 as overweight (BMI 25–29.9) and ≥6 as obesity (BMI ≥ 30). This classification was used to describe the students´ Body Percept.

#### 2.3.2. Desired Body Image

The Desired Body Image corresponded to the second selected silhouette, the one the participants considered to best represent the Body Image they would like to have. This variable was also linked to the BMI categories by using the number from 1–9 assigned to each silhouette. The cut points were: 1 as low weight (BMI < 18.5), 2–4 as standard weight (BMI 18.5–24.9), 5 as overweight (BMI 25–29.9) and ≥6 as obesity (BMI ≥ 30) [21]. This classification was used to describe the students´ Desired Body Image.

#### 2.3.3. Body Dissatisfaction

**Figure 1:**
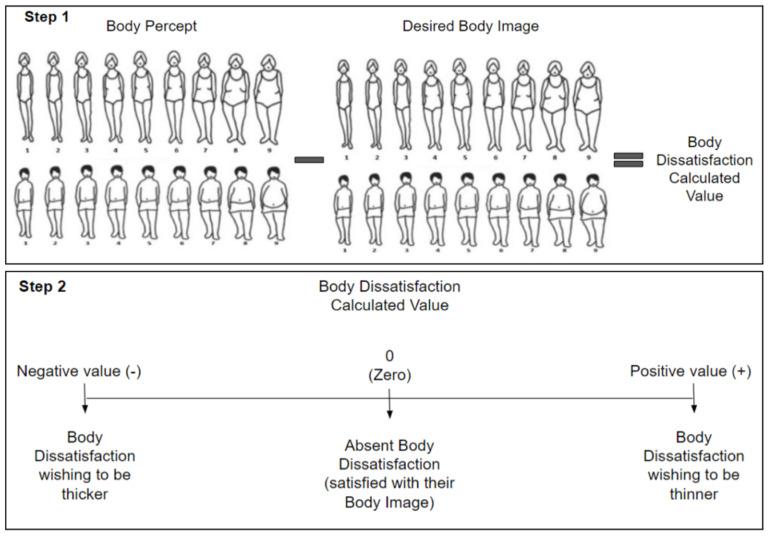
Body Dissatisfaction Assessment.

To define the presence of Body Dissatisfaction the discrepancy between Body Percept and Desired Body Image was calculated by using the number from 1–9 assigned to each selected silhouette (Figure 1, Step 1). The result from the calculated discrepancy was classified in the following categories: <0 (negative value) = Body Dissatisfaction, wishing to be thicker; 0 = Absent Body Dissatisfaction (satisfied with their Body Image); >0 (positive value) = Body Dissatisfaction, wishing to be thinner [20] (Figure 1, Step 2). This variable was also dichotomized by the following cut points: 0 meaning Absent Body Dissatisfaction and ≠0 meaning Present Body Dissatisfaction.

#### 2.3.4. Distorted Body Image

**Figure 2:**
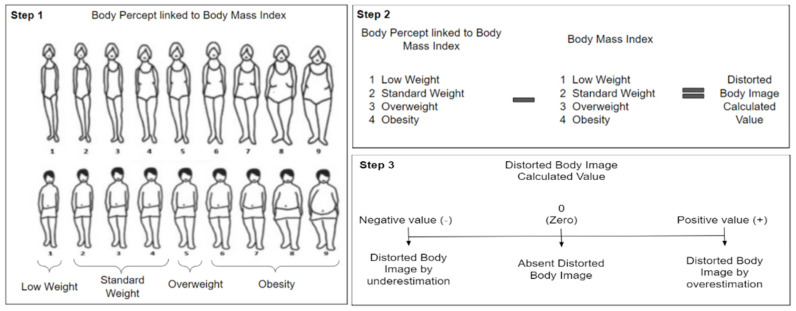
Distorted Body Image Assessment.

To assess Distorted Body Image the Stunkard´s Silhouette for Body Percept and the participant´s BMI were used. BMI was calculated by using self-reported weight and height. Both variables were classified according to the BMI categories and a number was assigned to each category: 1-low weight, 2-standard weight, 3-overweight and 4-obesity (Figure 2, Step 1). Then, the difference between the number assigned to Body Percept and the one assigned to the BMI was calculated (Figure 2, Step 2), leading to the following categories: <0 (negative value) = Distorted Body Image, by underestimation, 0 =Absent Distorted Body Image, >0 (positive value) =Distorted Body Image, by overestimation [4] (Figure 2, Step 3). This variable was also dichotomized by the following cut points: 0 meaning Absent Distorted Body Image and ≠0 meaning Present Distorted Body Image.

It was not possible to report Distorted Body Image in 2017 due to the lack of response in self-reported weight and height.

### 2.4. Disordered Eating Behaviors

Disordered Eating Behaviors were measured through the Brief Disordered Eating Behaviors Questionnaire (BDEBQ) which is an instrument developed and validated for the Mexican population [14]. It evaluates the following behaviors to lose weight in the three months prior to the application of the questionnaire: worrying about gaining weight, the practice of binge eating, feeling a lack of control when eating, restrictive eating behaviors (diets, fasts, exercise abuse and use of weight loss pills) and purgative behaviors (self-induced vomiting, use of laxatives, diuretics and enemas). It consists of 11 items on a Likert scale from 0-never or almost never to 3-very frequently (more than twice a week) and the total score goes from 0 to 33, by which the following categories are displayed: <7 Absent Disordered Eating Behaviors (or no risk for Eating Disorders), 7–10 Moderate Disordered Eating Behaviors (or moderate risk for Eating Disorders) and ≥11 High Disordered Eating Behaviors (or high risk for Eating Disorders). This variable can also be dichotomized by the following cut points: <7 Absent Disordered Eating Behaviors and ≥7 Present Disordered Eating Behaviors.

### 2.5. Statistical Analysis

In this study, descriptive statistics were used to describe age, sex, scholarly year, Body Percept and Desired Body Image of the participants. Prevalence for Disordered Eating Behaviors, Body Dissatisfaction and Distorted Body Image were calculated for each given year; changes in trends of prevalence of each variable were also analyzed for the six years, using a chi square for trend test.

Each year comparisons between Body Dissatisfaction and Distorted Body Image with Disordered Eating Behaviors were performed through Chi square tests.

Stratified Chi Square Tests between Body Dissatisfaction and Disordered Eating Behaviors were performed for each year of study, each one stratified for a specific sociodemographic or academic variable (age, sex or scholarly year).

Finally, a logistic regression for Disordered Eating Behaviors, adjusted by age, sex, Body Dissatisfaction and Distorted Body Image, was performed per year.

For all the analyses we used IBM SPSS Statistics for Windows, version 23.0 (IBM Corp., Armonk, NY, USA).

## 3. Results

### 3.1. Descriptive Analysis

During the six years of study, an approximate sample of 200–300 participants per year was obtained, excepting 2019, where there was greater participation (n = 319), and 2022, being the year with the lowest response (n = 174).

We observed that participation was concentrated mainly in women and those who were studying in their first two school years; with a median age of 21 (Table 1).

Regarding the Body Image-related variables (Table 2), we observed that the students mainly perceived themselves as having standard weight; the prevalence of this category was the highest in 2017 and decreased in 2018. After this year, the prevalence remained constant. Low weight in Body Percept increased slightly over the years, except for 2022 when it decreased. On the other hand, most of the participants desired a standard weight Body Image. Nevertheless, the percentage of low weight Desired Body Image rose each year.

In addition, more than half of the sample presented Body Dissatisfaction, wishing to be thinner. As for Distorted Body Image, most of those who presented it overestimated their weight. The prevalence of students who had both BD and DBI was over 30% every year. Moderate Disordered Eating Behaviors were more prevalent than high DEBs, although high DEBs increased over the years, almost doubling in prevalence from 2017 to 2022.

**Figure 3:**
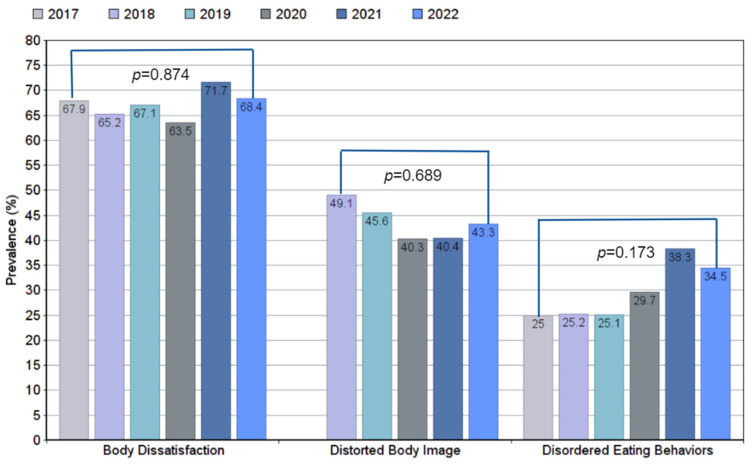
Chi square for trend test. Changes in the prevalence of Body Dissatisfaction, Distorted Body Image and Disordered Eating Behaviors in university students from 2017 to 2022.

Figure 3 shows the changes in prevalence of BD, DBI and DEBs from 2017 to 2022. Even though the changes during the six years period were not statistically significant, both BD and DEBs remained present in more than 60% and 25% of our sample, respectively; showing their highest peak in 2021. Whether there was significance in the changes in trends between 2020 and 2021 was also analyzed, since this represented the pre- and post-quarantine period, but there was no statistical significance. Despite the fact that DBI decreased over the years (except for 2022 where it rose a little), it remained present in more than 40% of the students.

### 3.2. Associations between Body Image-Related Variables and Disordered Eating Behaviors

During each of the six years of study, DEBs showed significant associations with the presence of BD and DBI (Table 3). The probability of having DEBs was 3.6 to 6.9 times higher in those with Body Dissatisfaction; this probability rocketed in 2022, where it was shown to be almost 16 times higher in comparison to those who did not have BD. The probability of presenting DEBs was 1.9 to 5.1 times higher in those who had Distorted Body Image than in those who did not have it. As for the students who presented both BD and DBI, there was a statistically significant probability 2.2 to 4 times higher for presenting DEBs during the six years of study.

We could appreciate that during the six years of study, both age groups had a higher probability of developing Disordered Eating Behaviors if they had Body Dissatisfaction. However, the probability was even higher in older participants (except for 2020 and 2022, where the younger group proved to have a higher probability). Regarding sex, women showed statistically significant associations yearly; for men, it was only significant during 2017. Therefore, we could say that women had 4.5 to 8.5 times more probability of having DEBs if they had BD, except for 2022, where BD appeared to be one of protection (OR: 0.55, *p* ≤ 0.001). Furthermore, during four of the six years of the study, those in the first school year showed the highest probability of developing DEBs in the presence of BD (OR: 10.9–33), except for 2019 and 2021, where the probability was higher in second and third years, respectively (Table 4).

### 3.3. Adjusted Models

Lastly, logistic regressions were performed between DEBs and BD, DBI, sex, and age (Table 5). It was found that during the six years of study, students with Body Dissatisfaction had a statistically significant, 3 to almost 13 times, greater probability of presenting DEBs, compared to those who were satisfied with their Body Image, adjusted with the presence of Distorted Body Image, sex and age (aOR: 3.75, *p* ≤ 0.001-aOR: 12.7, *p* = <0.001). Distorted Body Image only showed statistical significance in 2019 (aOR: 2.05, *p* = 0.014). Regarding sex, women only showed significant associations during 2018, which meant that within the logistic regression, being a woman lost significance in five of the six years of study.

## 4. Discussion

The main objective of this study was to analyze the changes in the prevalence of Body Dissatisfaction, Distorted Body Image and Disordered Eating Behaviors in university students over a six-year period. The associations between the conditions were also reported. It was found that the presence of BD and DBI was associated with the presence of DEBs.

Regarding sociodemographic data, each year samples mainly consisted of female students, with a frequency ranging from 64.5–72.5%, and those in early semesters, with frequencies ranging from 40.8–64.3%. Sex frequencies were similar to the ones reported in another study in Mexican university students, in which the participation of female students was higher [22], and it also matched the sex and school year distribution of the students during the six years in the University of our samples.

As for Body Image-related variables, Body Percept did not vary much among the six samples. Throughout the period, more than half of the students perceived themselves as standard weight, followed by obesity; this did not match another study in Mexican public university students [23]. Instead, they mainly perceived themselves as overweight or obese (39.6%) [23]. We think that the differences between studies may have been due to samples coming from differing socioeconomic backgrounds; individuals with a high socioeconomic status are described to have better access to health information and to be more weight-conscious [24]. Even though the number of students who perceived themselves with low weight slightly increased during 2019 and continued increasing until it doubled in the sample of 2021, it showed its lowest prevalence in 2022 and remained the least selected category of Body Percept during the six-year period, which agreed with the report for the Mexican population [23].

Across the six samples, Body Dissatisfaction was found in more than half of the students, wishing for a thinner figure; this result was similar to the results obtained in Spanish and Mexican populations with a percentage of 64% and 78%, respectively [25]. It also matched Venezuelan population prevalence (80–84.6%) [26].

The prevalence for moderate DEBs (17.6–26.4%) were higher than for high DEBs (6.7–14.4%) in all six samples; similar prevalence was observed in Bachelor students, in which moderate DEBs had a 25.1% prevalence and high DEBs had a 11.6% prevalence [27].

Throughout the six years of study BD and DEB maintained prevalence above 60% and 25%, respectively. DEB prevalence (25–38.3%) was higher than those reported by a university in the state of Hidalgo, Mexico (17.1%) [28], and lower than that reported for Bachelor in Nutrition students (36.7%) [27]. The differences found in the prevalence of our samples compared to others reported for Mexican university students might have been due to the fact that the risk of presenting DEBs has been reported to vary among careers and school semesters [27,29]. Therefore, the prevalence could change according to the careers that each university offers studies towards and which were included in the analysis, as well as the semester distribution of samples. Although, the increase in prevalence during the study period did not show statistical significance, high prevalence was found and did not seem to decrease. One study showed an upward trend for DEBs over the years, both for moderate and high CAR, coinciding with our results [30].I It is important to mention that this study was carried out more than 10 years ago and no recent studies in similar populations to ours have reported DEB prevalence changes classifying them by severity, over the years. We can also appreciate that the year with the highest prevalence for both, Body Dissatisfaction and Disordered Eating Behaviors was 2021, which then lowered by 2022. We believe that this may have been related to quarantine because of the COVID-19 pandemic; as social distancing has been associated with higher levels of Body Dissatisfaction [31,32], appearance concerns [33], increased levels of exercise as a compensatory measure to lose weight, and binge eating [32].

This study showed that the probability to present DEBs was higher in the presence of BD during the six years of study; this matched a report on Nursing university students, where the prevalence of DEBs was higher in those who were not satisfied with their Body Image [34]. Another study showed that the presence of BD predisposed the presence of DEBs, finding a moderate DEB prevalence of 86% and 74.4% for high DEBs in students who desired a slenderer figure [20]. There was a higher probability for DEBs in those who had Distorted Body Image, as shown in another study on young women [35]. As for the probability for DEBs in students who had Body Dissatisfaction and Distorted Body Image at the same time there was statistical significance in the six studied samples. No study was found that had previously studied this association; however, the associations were known separately [10,36].

Regarding the association between DEBs and BD, stratified by sociodemographic and academic data, we found that even though both age groups had significant associations, for most of the evaluated years, the association was higher in the older group; something similar was found in a study on adolescents [37]. Women showed greater associations in almost all of the assessed years; in the literature, it has been reported that restrictive diets and methods are more prevalent among women and they tend to be less satisfied with their bodies [38]. There were significant associations for the probability of developing DEBs in the presence of Body Dissatisfaction during almost all the years of study, mainly in the first school year. The specific association between DEBs and BD stratified by school year has not been studied previously; however, the association between BD and school year was studied in [29] (REF). The transition during the first year of university generated vulnerability for the presence of DEBs in students in [39].

As for the logistic regressions between DEBs with BD, DBI, age, and sex, Body Dissatisfaction maintained significant associations during the six years; this led to the conclusion that Body Dissatisfaction was the primary factor associated with the development of DEBS, as reported in the literature [11]. Distorted Body Image, sex and age lost significant associations; this might have been because, although significant associations with these variables were found separately when studied or observed, together in a statistical model, the way in which they behave changed.

The main strength of our study was that we compared the changes in prevalence of DEBs and Body Image-related variables and their associations across six years in a sample of university students. The period of time covered by our study was very valuable, since it included measurements from before, during, and after social isolation due to the COVID-19 pandemic; a situation that led to academic, health, economic and social changes. In addition, the assessment of various Body Image-related variables offered a complete overview of the phenomenon; hardly seen in other publications. During the complete study period no modifications to the instruments were made, which was another strength of this project.

The self-reported weight and height may seem a limitation of our study; nevertheless, studies have concluded that it is a valid assessment [40,41].

Although we invited all students to participate in the study, only those who agreed were included. This could result in selection bias, in that only those who felt or perceived themselves as healthy and/or comfortable with the questionnaire variables participated. Another possible limitation is that our sample size may have been too small, as our results showed wide OR confidence intervals, maybe to the varied response rate across the years (41 to 88%).

Regarding the sample characteristics, we could not affirm that the participants represented average university students from Mexico City since it was carried out at a private university.

There is no consensus on definitions or standardized terms in clinical and research settings. It is noteworthy that since there are different ways to refer to the same concept, it would be ideal to have a consensus on how to name these variables. For example, Distorted Body Image is sometimes reported as “Misperception” and also as “Underestimation” (which is just one category of the variable); this complicates the comparison of the results.

The Stunkard instrument was created and validated in the 1980s [10]; its relationship with BMI categories was validated in the 2000s [21]. Although it is still widely used, we believe it would be important to reassess the instrument and its relationship with BMI categories as silhouettes may not clearly represent all body compositions, for example, those with an image thicker than silhouette number 9. So, perhaps, it would be optimal to specify that those who consider their silhouette to be thicker than 9 should still select this silhouette. This simple clarification could reduce the lack of representation that these people usually suffer. It can be seen that only one of the silhouettes represents the “overweight” category, and everything else in that category is considered obese. This leads us to question whether its association with the BMI categories could be biased by the fat-phobic culture, the resignification and elimination of which is currently being sought.

We also suggest that future investigations include other nutritional and/or mental health characteristics.

## 5. Conclusions

The prevalence of Body Image-related variables was high and steady throughout the studied six years in university students, even though the participants were not the same each year of study. In 2021, especially, the prevalence of Body Dissatisfaction and Disordered Eating Behaviors were higher due to the impact of the COVID-19 quarantine.

We recommend regular screening for alterations in Body Image-related variables and eating behaviors, mainly in women and the young population. It is important to stop normalizing unhealthy beauty stereotypes, the diet culture and the belief that one’s worth is defined by thinness; as all of these beliefs and conducts may lead to Body Dissatisfaction, Distorted Body Image, Disordered Eating Behaviors and Eating Disorders.

Preventing the evolution, and promotion, of the assessment of these issues is a public health necessity as they usually remain undiagnosed, while their prevalence are increasing. Their impact on physical and mental health has already been seen, so we must no longer consider them as merely a fashion or as something outside the competence of health personnel.

## Figures and Tables

**Table 1:** General characteristics of each year’s sample (2017–2022).

Variable	2017 n = 296 n (%)	2018 n = 282 n (%)	2019 n = 319 n (%)	2020 n = 219 n (%)	2021 n = 201 n (%)	2022 n = 174 n (%)
Age *	21 (18–30)	21 (18–27)	21 (18–28)	20 (18–29)	21 (18–30)	20 (18–27)
Sex						
Woman	191 (64.5)	181 (64.2)	225 (69)	142 (64.8)	146 (72.6)	124 (71.3)
Man	105 (35.5)	101 (35.8)	99 (31)	77 (35.2)	55 (27.4)	50 (28.7)
School Year						
1°& 2°	121 (40.8)	142 (50.4)	155 (48.6)	119 (54.3)	106 (52.7)	112 (64.3)
3°& 4°	126 (42.6)	97 (34.4)	115 (36)	66 (30.1)	69 (34.3)	44 (25.2)
5°& 6°	49 (16.5)	43 (15.3)	51 (15.4)	34 (15.5)	26 (13)	18 (10.3)

* Results reported in median (min.-max).

**Table 2:** Prevalence of Body Image-related variables in university students (2017–2022).

Variable	2017 n = 296 n (%)	2018 n = 282 n (%)	2019 n = 319 n (%)	2020 n = 219 n (%)	2021 n = 201 n (%)	2022 n = 174 n (%)
**Body Percept**						
Low weight	4 (1.4)	4 (1.4)	6 (1.9)	5 (2.3)	6 (3)	1 (0.6)
Standard weight	190 (64.2)	150 (53.2)	182 (57.1)	121 (55.3)	113 (56.2)	99 (56.9)
Overweight	43 (14.5)	57 (20.2)	50 (15.7)	39 (17.8)	34 (16.9)	25 (14.4)
Obesity	59 (19.9)	71 (25.2)	81 (25.4)	54 (24.7)	48 (23.9)	49 (28.2)
**Desired Body Image**						
Low weight	4 (1.4)	5 (1.8)	6 (1.9)	4 (1.8)	6 (3)	6 (3.4)
Standard weight	257 (86.8)	237 (84)	270 (84.6)	184 (84)	177 (88.1)	146 (83.9)
Overweight	32 (10.8)	31 (11)	34 (10.7)	23 (10.5)	18 (9)	17 (9.8)
Obesity	3 (1.0)	9 (3.2)	9 (2.8)	8 (3.7)	0 (0)	5 (2.9)
**Body Dissatisfaction**						
Absent	95 (32.1)	98 (34.8)	105 (32.9)	80 (36.5)	57 (28.4)	55 (31.6)
Present (wishing to be thinner)	167 (56.4)	157 (55.7)	178 (55.8)	112 (51.1)	124 (61.7)	106 (60.9)
Present (wishing to be thicker)	34 (11.5)	27 (9.6)	36 (11.3)	27 (12.3)	20 (10)	13 (7.5)
Distorted Body Image *						
Absent	-	136 (50.9)	161 (54.4)	125 (59.5)	115 (59.6)	93 (56.7)
Present (by underestimation)	-	12 (4.5)	22 (7.4)	12 (5.5)	8 (4.1)	10 (6.1)
Present (by overestimation) **BD + DBI**	-	119 (44.6)	113 (38.2)	73 (36.3)	70 (36.3)	61 (37.2)
Absent	-	183 (65.1)	200 (62.7)	149 (67.1)	132 (65.7)	112 (64.4)
Present	-	98 (34.9)	119 (37.3)	73 (32.9)	69 (34.3)	62 (35.6)
**Disordered Eating Behaviors**						
Absent	222 (75)	211 (74.8)	239 (74.9)	154 (70.3)	124 (61.7)	114 (65.5)
Moderate	52 (17.6)	52 (18.4)	57 (17.9)	42 (19.2)	53 (26.4)	35 (20.1)
High	22 (7.4)	19 (6.7)	23 (7.2)	23 (10.5)	24 (11.9)	25 (14.4)

* The total n in this variable varies from the total n of the sample, due to participants′ incomplete data in self-reported weight and/or height; the table shows the valid percentages. BD = Body Dissatisfaction. DBI = Distorted Body Image.

**Table 3:** Association between Body Dissatisfaction and Distorted Body Image with Disordered Eating Behaviors (2017–2022).

Variable	2017		2018		2019		2020		2021		2022	
	OR	95% CI	OR	95% CI	OR	95% CI	OR	95% CI	OR	95% CI	OR	95% CI
**Body Dissatisfaction**												
Present	6.2	2.7–14.3 *	6.9	3.0–15.8 *	3.6	1.8–7.0 *	3.9	1.9–8.2 *	5.6	2.4–12.7 *	15.9	4.7–53.8 *
Absent	1		1		1		1		1		1	
**Distorted Body Image**												
Present	-	-	2.2	1.2–3.9 *	2.7	1.5–4.7 *	1.9	1.–3.4 *	2.3	1.3–4.3 *	3.3	1.7–6.4 *
Absent	-	-	1		1		1		1		1	
**BD + DBI**												
Present	-	-	2.2	1.3–3.9 *	3.3	1.9–5.5 *	3.0	1.6–5.5 *	2.6	1.4–4.8 *	4.0	2.0–7.8 *
Absent	-	-	1		1		1		1		1	

* Statistically significant *p* value (*p* ≤ 0.001). BD = Body Dissatisfaction. DBI = Distorted Body Image.

**Table 4:** Associations between Disordered Eating Behaviors and Body Dissatisfaction stratified by age, sex and school year (2017–2022).

	2017		2018		2019		2020		2021		2022	
	OR	95% CI	OR	95% CI	OR	95% CI	OR	95% CI	OR	95% CI	OR	95% CI
**Age**												
<21	4.7	1.3–16.8 *	5.0	1.0–24.6 *	2.9	1.2–6.9 *	5.6	1.5–20.2 *	4.9	1.5–16.2 *	34.9	4.5–269.9 *
≥21	7.5	2.5–22.3 *	7.6	2.8–20.2 *	5.1	1.7–15.3 *	3.5	1.4–8.8 *	6.2	2.0–19.2 *	7.2	1.5–34.4 *
**Sex**												
Women	7.0	2.6–18.6 *	8.5	3.2–22.9 *	4.5	2.0–10.2 *	5.1	2.1–12.6 *	6.3	2.4–16.3 *	0.5	0.4–0.6 *
Men	5.0	1.0–23.2 *	3.8	0.7–18.2	2.0	0.6–6.8	2.3	0.6–7.9	3.9	0.7–19.7	3.7	0.9–15.7
**School Year**												
1°	12.2	0.6–218.5 *	13.3	1.6–106.6 *	2.0	0.7–5.5	10.9	1.3–90.5 *	7.0	1.4–34.3	33	4.1–262.9 *
2°	3.3	0.8–14.0	10.4	1.2–88.1 *	13	0.7–232.8 *	4.2	0.8–21.2	3.2	0.7–14.0	5.1	0.5–47.9
3°	7.9	0.9–65.5 *	10.1	1.1–86.8 *	5.0	1.0–24.2 *	1.9	0.5–7.5	12.8	1.3–118.3 *	8.3	0.7–89.4
4°	9.8	1.1–81.4 *	2.8	0.5–14.6	3.2	0.6–17.0	7.0	0.6–75.7	3.5	0.3–33.7	0.6	0.4–0.9
5°	10.0	0.9–104.4	1.2	0.0–38.2	4.2	0.4–39.4	4.0	0.1–120.7	8.0	0.5–127.9	0.5	0.2–1.1
6°	3.6	0.3–37.4	3.6	0.5–21.9	6.2	0.2–146.7	2.8	0.4–16.9	1.5	1.0–2.4	0.7	0.5–1.1

* Statistically significant *p* value.

**Table 5:** Association between Disordered Eating Behaviors and Body Image-related variables. Logistic regression models adjusted by sociodemographic data.

Independent Variables	Disordered Eating Behaviors											
	2017		2018		2019		2020		2021		2022	
	aOR	95% CI	aOR	95% CI	aOR	95% CI	aOR	95% CI	aOR	95% CI	aOR	95% CI
**Body Dissatisfaction**												
Absent		1		1		1		1		1		1
Present	6.2	2.7–14.3 *	5.6	2.3–13.4 *	2.8	1.3–5.8*	3.7	1.7–8.1 *	4.1	1.7–9.7 *	12.7	3.6–44.7 *
**Distorted Body Image**												
Absent	-	-		1		1		1		1		1
Present	-	-	1.2	0.6–2.2	2.0	1.1–3.6 *	1.3	0.6–2.5	1.6	0.8–3.1	1.9	0.9–4.0
**Sex**												
Woman	1.7	0.9–3.3	3.3	1.6–6.8 *	1.2	0.7–2.3	1.9	0.9–3.8	1.9	0.9–4.1	0.9	0.3–2.0
Man		1		1		1		1		1		1
**Age**	1.0	0.9–1.2	1.0	0.9–1.2	0.9	0.8–1.1	1.2	1.0–1.5 *	1.0	0.8–1.2	0.9	0.7–1.0

aOR = Adjusted Odds Ratio. * Statistically significant *p* value.

## Data Availability

Not applicable.

## Abbreviations

BD	Body Dissatisfaction
DBI	Distorted Body Image
DEBs	Disordered Eating Behaviors
EDs	Eating Disorders
BMI	Body Mass Index
